# Supplementary material for: The role of mixed vibronic Qy-Qx states in green light absorption of light-harvesting complex II
Source: Nat Commun. 2020 Nov 26;11:6011. doi: 10.1038/s41467-020-19800-y (PMC7691517; doi:10.1038/s41467-020-19800-y)
Supplement: Supplementary file 1 — Supplementary Information [file 41467_2020_19800_MOESM1_ESM.pdf]

Supplementary Information

The role of mixed vibronic Q_y-Q_x states in green light absorption of light-harvesting complex II

Eric A. Arsenault[1,2,3], Yusuke Yoneda[1,2,3], Masakazu Iwai[3,4], Krishna K. Niyogi[3,4], and Graham R. Fleming[1,2,3*]

[1]Department of Chemistry, University of California, Berkeley, CA 94720, USA

[2]Kavli Energy Nanoscience Institute at Berkeley, Berkeley, CA 94720, USA

[3]Molecular Biophysics and Integrated Bioimaging Division, Lawrence Berkeley National Laboratory, Berkeley, CA 94720, USA

[4]Department of Plant and Microbial Biology, University of California, Berkeley, CA 94720, USA

[*]grfleming@lbl.gov

Supplementary Table 1. Fit Parameters for the peak amplitude dynamics provided in Fig. 2c. Fig 2f, and Fig. 3c

PA Component	$\omega_{det.}$ (cm^{-1})[a]	A_0	A_1	τ_1 (fs)[b]	ω_1 (cm^{-1})[c]	φ_1 (π)[d]	A_2	τ_2 (fs)[e]	ω_2 (cm^{-1})	A_3	τ_3 (fs)
⊥	1690	0.42	--	--	--	--	--	--	--	--	--
ǁ		0.17	-0.01	140±30	111±7	0.7±0.2	-0.001	--	240±20	0.01	330±120
⊥	1680	0.01	--	--	--	--	--	--	--	-0.01	600±200
ǁ		0.19	-0.01	140±30	90±10	0.8±0.3	--	--	--	--	--
⊥	1670	-0.93	--	--	--	--	--	--	--	-0.01	600±200
ǁ		-0.56	-0.01	190±30	106±5	1.1±0.2	-0.001	--	240±10	--	--

[a]Excitation frequency fixed at 17800 cm^{-1}.

[b,c,d]Errors indicate 1σ intervals.

[e]Damping time unable to be fit precisely without smaller timesteps past 625 fs, but are likely >600 fs.

The fit function employed is given by:

$$f(t) = A_0 + A_1 \cos(2\pi\omega_1 t + \varphi_1)\, e^{-t/\tau_1} + A_2 \cos(2\pi\omega_2 t)\, e^{-t/\tau_2} + A_3 e^{-t/\tau_3}$$

convoluted with the instrument response function.

Supplementary Table 2. Fit Parameters for the frequency distribution dynamics along the excitation provided in Fig. 3g

PA Component	$\omega_{det.}$ (cm^{-1})	A_0 (cm^{-1})	A_1 (cm^{-1})	τ_1 (fs)[a]	ω_1 (cm^{-1})[b]	φ_1 (π)[c]	A_2 (cm^{-1})	τ_2 (fs)[d]	ω_2 (cm^{-1})
∥	1670	17821.70	-1.12	250±50	92±6	1.33±0.19	-0.14	--	240±20

[a,b,c]Errors indicate 1σ intervals.

[d]Damping time unable to be fit precisely without smaller timesteps past 625 fs, but are likely >600 fs.

The fit function employed is given by:

$$f(t) = A_0 + A_1 \cos(2\pi\omega_1 t + \varphi_1)\, e^{-t/\tau_1} + A_2 \cos(2\pi\omega_2 t)\, e^{-t/\tau_2}.$$
